# The MTHFR C677T genetic susceptibility and its role in risk prediction for type 2 diabetic nephropathy in Northern China

**DOI:** 10.3389/fendo.2026.1749477

**Published:** 2026-01-21

**Authors:** Junli Song, Juanjuan Zhang, Qian Guo, Qiang Zhao

**Affiliations:** 1 Department of Pharmacy, Second Hospital of Shanxi Medical University, Taiyuan, Shanxi, China; 2 School of Pharmacy, Shanxi Medical University, Taiyuan, Shanxi, China

**Keywords:** diabetic nephropathy, homocysteinemia(HCY), methylenetetrahydrofolate reductase (MTHFR), nomogram, risk prediction

## Abstract

**Objective:**

Diabetic nephropathy is a diabetes-induced chronic kidney disease characterized by pathological changes that may involve the entire renal structure and can progress to end-stage renal disease, thereby substantially impairing patients’ quality of life. The MTHFR C677T gene polymorphism has been closely associated with the development of diabetic nephropathy through its regulatory effect on homocysteine levels. Evidence suggests that both the MTHFR C677T genetic variant and other clinical or environmental risk factors may interactively contribute to disease susceptibility. This study investigated the genetic predisposition conferred by MTHFR C677T and its interaction with modifiable risk factors among patients with diabetic nephropathy in northern China, aiming to establish a region-specific risk prediction model for improved early identification and prevention.

**Material and methods:**

From January 2018 to 2024, a total of 397 patients with type 2 diabetes were selected from the Second Hospital of Shanxi Medical University. Among them, 153 cases were diagnosed as diabetic nephropathy group (DN group), and the remaining 244 cases were control group (N-DN group). The clinical data of these patients were extracted from electronic medical records. The biochemistry index, including homocysteine (Hcy) were collected from the hospital laboratory test system and the polymorphism of MTHFR C677T gene was analyzed by polymerase chain reaction (PCR) test. The risk prediction model of diabetic nephropathy was established by Logistic regression.

**Results:**

MTHFR C677T gene polymorphism was closely related to diabetic nephropathy. The proportion of 677TT genotype (57.52%) and the homocysteine concentration in DN group were significantly higher than those in N-DN group, and the circulating homocysteine concentration in 677TT genotype (17.29 ± 8.95 μmol/L) was significantly higher than that in 677CT genotype (13.08 ± 6.20 μmol/L) and 677CC genotype (12.65 ± 4.35 μmol/L) (P < 0.001). The carriers of MTHFR677CT and MTHFR677TT genotypes could increase 3.298-fold and 12.713-fold risk of having DN respectively compared with the carriers of 677CC genotype.In addition, the diabetic peripheralangiopathy, the diabetic retinopathy, triglyceride(TG), HOMA-IR, HCY(16-30μmol/L), HCY(>30μmol/L), Blood urea nitrogen(BUN), urinary microalbumin creatinine ratio (ACR) were independent risk factors for diabetic retinopathy (OR = 2.462, 4.572, 1.548, 1.133, 1.571,1.379,1.254,1.003). The above eight factors constitute the Nomogram risk prediction model of DN with good test performance and discriminant ability.

**Conclusion:**

MTHFR C677T genotype, Hcy, the diabetic peripheralangiopathy, the diabetic retinopathy, triglyceride(TG), HOMA-IR, Blood urea nitrogen(BUN), urinary microalbumin creatinine ratio (ACR)diabetic retinopathy were independent factors for diabetic nephropathy, which would help to identify the risk of diabetic nephropathy and provide the basis for the development of diabetic nephropathy control strategies and treatment measures.

## 1 Introduction

Diabetes is a prevalent chronic non-communicable disease characterized by persistent hyperglycemia resulting from defects in insulin secretion, insulin action, or both. Over recent decades, rapid economic development, lifestyle changes, and population aging have contributed to a sharp increase in the incidence of diabetes in China. Currently, diabetes has become a major public health challenge, posing a serious threat to human health and ranking alongside cardiovascular and cerebrovascular diseases and malignant tumors as one of the leading causes of morbidity and mortality among the Chinese population (1, 2). National epidemiological surveys indicate that the prevalence of diabetes among adults in China exceeds 10%, with a significantly rising trend observed in both urban and rural areas, placing substantial strain on healthcare systems and social resources.

Diabetic nephropathy (DN) is a serious microvascular complication of diabetes mellitus and represents a leading cause of increased morbidity and mortality, significantly contributing to reduced life expectancy in individuals with type 2 diabetes (3–5). It typically develops after several years of poorly controlled hyperglycemia and is characterized by progressive albuminuria, a declining glomerular filtration rate, and structural renal abnormalities such as thickening of the glomerular basement membrane and mesangial expansion. Approximately 20% to 40% of individuals with diabetes in China are affected by diabetic kidney disease, which has emerged as the leading cause of chronic kidney disease (CKD) and end-stage renal disease (ESRD) (6–8). Homocysteine (Hcy) is a sulfur-containing, non-proteinogenic amino acid derived from the metabolism of methionine. Hyperhomocysteinemia (HHcy)—defined as elevated levels of homocysteine in the blood—has emerged as an independent risk factor for vascular endothelial damage and oxidative stress, both of which play pivotal roles in the onset and progression of diabetic kidney disease. HHcy is particularly prevalent among patients with type 2 diabetes and impaired renal function.Methylenetetrahydrofolate reductase (MTHFR) serves as a pivotal enzyme in Hcy metabolic pathways. Evidence from prior studies indicates that the C677T polymorphism in the catalytic domain of the MTHFR gene can impair enzymatic activity, resulting in disrupted Hcy metabolism, intracellular accumulation of Hcy, and consequently elevated plasma Hcy levels (9, 10). Numerous clinical and molecular studies have demonstrated a significant association between the MTHFR C677T variant and an elevated risk of type 2 diabetic nephropathy (11, 12). These findings underscore the importance of integrating genetic screening with metabolic assessment in evaluating individual susceptibility to DN, potentially facilitating earlier intervention and personalized management strategies.

Shanxi Province, with a total area of 156,700 km² and a population of 37.02 million, is situated in northern China. Previous research has demonstrated that the genotype frequency of the MTHFR C677T polymorphism in this region reaches 80.41%. Furthermore, it has been established that despite dietary patterns conducive to lowering homocysteine levels, the high prevalence of hyperhomocysteinemia remains significantly associated with the MTHFR C677T genetic variant (13). Given these findings, a critical question arises: does the genetic susceptibility conferred by the MTHFR C677T polymorphism, in conjunction with its closely linked elevation in homocysteine levels, contribute to the onset and progression of diabetic nephropathy in this population?

Based on these findings, the present study was designed to address several critical questions regarding the role of the MTHFR C677T polymorphism in diabetic nephropathy. First, to determine whether the MTHFR C677T genetic variant, in conjunction with elevated homocysteine levels within the genetic background, contributes significantly to the onset and progression of diabetic nephropathy. Second, the potential pathogenic pathway linking the MTHFR C677T genetic variant, elevated homocysteine levels, and diabetic nephropathy was systematically investigated. Third, multiple factors associated with the risk of diabetic nephropathy were evaluated to identify potential predictive indicators and provide clinicians with valuable data for assessing DN-related risk factors. Finally, a nomogram-based risk prediction model for diabetic nephropathy was developed to provide an effective tool for predicting the development of DN in patients with diabetes.

## 2 Materials and methods

### 2.1 Sample size calculation

According to the literature survey, we knew that the incidence of diabetic nephropathy among Chinese patients with diabetes is 20%-40%, the proportion of the MTHFR 677TT gene in DN it is 51.2%. Based on an inspection power of 0.9 and confidence of 1-α=0.95, PASS15 software was used to calculate the sample size. The result showed that the sample size was 228. We assumed that the data unavailability rate was 20%. Thus the sample size N = 228/0.80 = 295 cases were required.

Additionally, in the study we aim to study the influencing factors of diabetic nephropathy. The sample size should be no less than 15–20 times the number of independent variables. According to the research plan, the number of independent variables is 18; the sample size of this part of the study should range from 270 to 360 cases.

### 2.2 Study population

In this retrospective study, 397 unrelated individuals (222 males and 175 females) who underwent MTHFR gene testing were enrolled from patients visiting the Second Hospital of Shanxi Medical University between January 2018 and December 2024, providing a sample size sufficient to meet the study’s research requirements.

Inclusion criteria were as follows: (i) participants meeting the diagnostic criteria for type 2 diabetes mellitus according to the Chinese Guidelines for the Prevention and Treatment of Diabetes Mellitus (2024 edition). Typical symptoms include polydipsia, polyuria, polyphagia, and unexplained weight loss. A diagnosis was confirmed if at least one of the following laboratory criteria was satisfied: fasting venous plasma glucose ≥ 7.0 mmol/L, 2-hour plasma glucose ≥ 11.1 mmol/L during an oral glucose tolerance test (OGTT), HbA1c ≥ 6.5%, or random venous plasma glucose ≥ 11.1 mmol/L in the presence of hyperglycemia-related symptoms. In asymptomatic individuals, diagnosis required either two abnormal results from different tests performed on the same blood sample or two separate elevated measurements obtained on different occasions (excluding random glucose values). All cases must have undergone differential diagnosis to exclude other forms of diabetes prior to classification as type 2 diabetes. (ii) Age of 18 years or older; (iii) non-immigrant status with continuous residence in Shanxi Province for at least two years; (iv) Han Chinese ethnicity with no first- or second-degree familial relationship among participants.

The exclusion criteria were as follows: (i) those who had taken folic acid tablets, or vitamin B/multivitamins supplements within the preceding 6 months; (ii) Individuals suffering from infectious conditions, cancerous growths, blood-related abnormalities, connective tissue disorders, endocrine disorders influencing homocysteine metabolism, as well as those diagnosed with congenital nephrotic syndrome.

Furthermore, according to the diagnostic criteria specified in the National Kidney Foundation/Kidney Disease Outcomes Quality Initiative (NKF/KDOQI) guidelines, a diagnosis of diabetic nephropathy was confirmed if any of the following criteria were met: (1) a documented history of diabetes mellitus, with exclusion of primary or secondary glomerular diseases and systemic conditions, and presence of either urinary albumin-to-creatinine ratio (UACR) ≥30 mg/g or 24-hour urinary albumin excretion rate ≥30 mg/24 h, with at least two abnormal results from three measurements collected during a 3- to 6-month period; (2) sustained reduction in estimated glomerular filtration rate (eGFR) <60 mL/min/1.73 m² for more than three months in patients with diabetes; or (3) renal biopsy findings consistent with diabetic nephropathy. Among the 397 study participants, 153 individuals who fulfilled these criteria were assigned to the case group (DN group), while the remaining 244 participants without evidence of nephropathy were categorized into the control group (N-DN group).

### 2.3 Demographic information and biochemistry index collection

As part of the patient’s hospital visits information, the basic demographic data of the participants (including gender, age, marital status, residence, smoking, alcohol consumption, family history, current medical history, past medical history, medication status, etc.) were collected from the hospital medical records system. In addition, the biochemistry index, including total cholesterol (TC), triglycerides (TG), low-density lipoprotein cholesterol (LDL-C), alanine aminotransferase (ALT), aspartate transaminase (AST), homocysteine (Hcy), Glycated hemoglobin (HbA1c), blood urea nitrogen (BUN), creatinine (Cr), urinary microalbumin (Alb), urinary microalbumin-to-creatinine ratio (ACR), etc, were collected from the hospital laboratory test system.

All staff has been specially trained in the early stage of the study, and the method of double inquiry and double input was adopted to ensure the authenticity and reliability of the data.

### 2.4 Genotyping of MTHFR polymorphism

Blood samples were collected from participants following an overnight fast of 8 to 14 hours. Approximately 3–5 mL of blood was drawn from the Participants antecubital’s vein. The blood was promptly transferred into EDTA anticoagulant tubes and gently inverted to ensure proper mixing, thereby minimizing the risk of coagulation or hemolysis. DNA was extracted from whole blood samples using a DNA extractionkit, according to the instructions (BaiO Technology Co,Ltd.).

**Figure 1:**
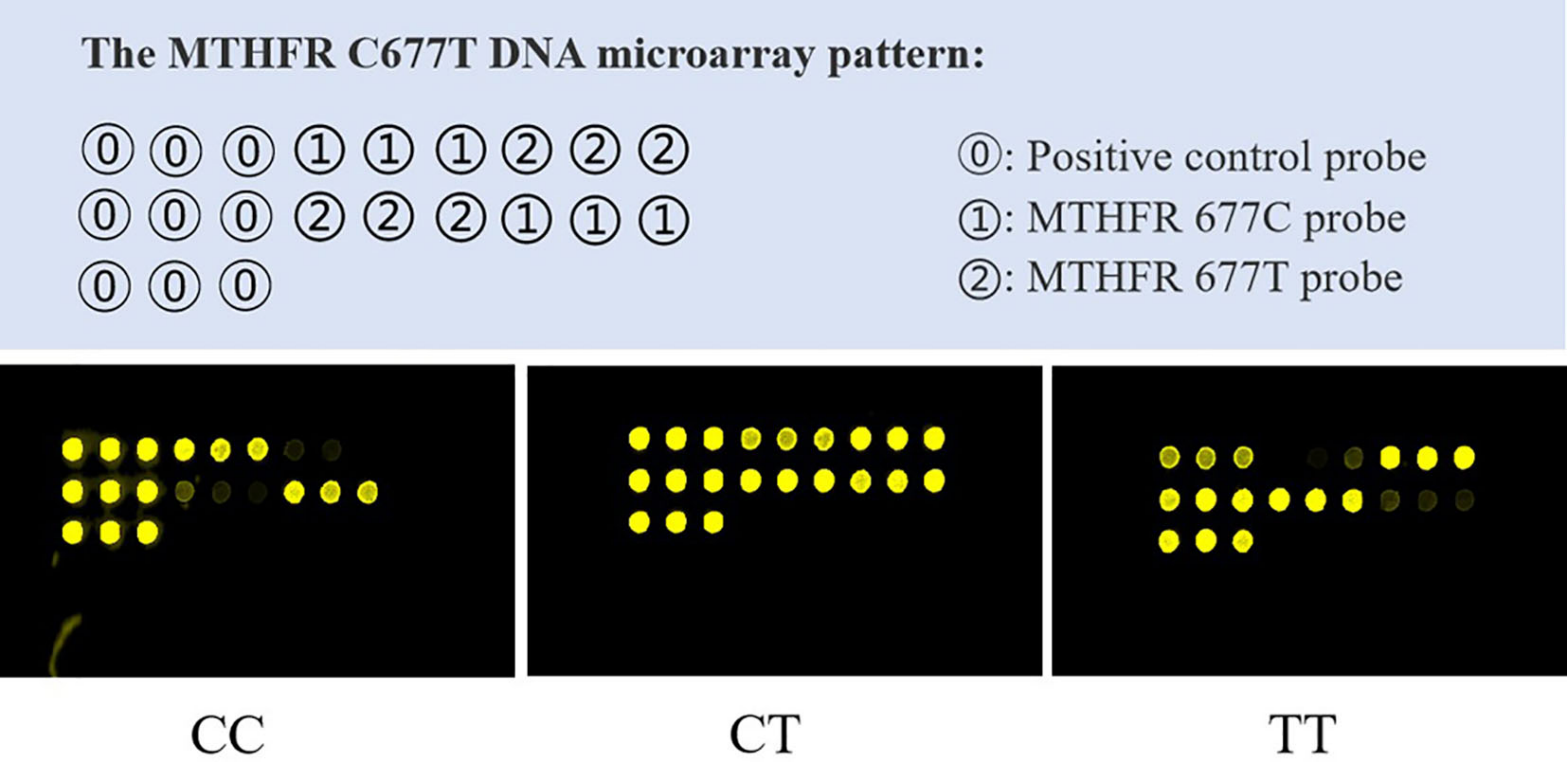
Microarray visualizations diagrams of the MTHFR C677T DNA.

Genotyping of MTHFR C677T polymorphisms was performed using PCR amplification and microarrays using commercially available kits (BaiO Technology Co., Ltd.). A 25μL PCR reaction mixture contained 20 ng DNA template and the recommended amounts of primers, dNTPs, and Taq DNA polymerase. The PCR thermocycling conditions were as follows: Predenaturing at 94°C for 5 minutes, followed by 35 cycles of 94°C for 25 seconds, 56°C for 25 seconds, and 72°C for 25 seconds, and a final extension at 72°C for 5minutes. The PCR products were then dispensed into a hybridization reaction chamber for hybridizing reactions. Based on the mutation from the wild type of MTHFR at position 677 from C to T, the MTHFR was divided into homozygous C allele (CC), heterozygous (CT), and homozygous T allele (TT) genotypes. Genotypes of MTHFR C677T were visualized by using the BaiO Array Doctor Version 2.0 software and BaiOBE-2.0 software according to the manufacturer’s protocol (BaiO Technology Co, Ltd.). The microarray visualizations diagrams of the MTHFR C677T were shown in **Figure 1**.

### 2.5 Statistical analyses

Statistical analyses were conducted using SPSS version 22.0 (SPSS Inc., Chicago, IL, USA). The representativeness of the study population was assessed through a Hardy-Weinberg equilibrium test. A p-value exceeding 0.05 indicated that the observed genotype distribution was consistent with the expectations under Hardy-Weinberg equilibrium.

A Shapiro-Wilk test was used to determine the distribution of the data. Normally distributed continuous variables are reported as mean ± standard deviation, while non-normally distributed data are presented as median ± interquartile range. The Kruskal-Wallis H test was employed to assess overall differences in homocysteine (Hcy) levels across genotype groups, and Dunn’s *post-hoc* test was applied for pairwise comparisons between the CT and TT groups relative to the CC group. Categorical variables are presented as frequency and percentage (n,%). The chi-square test (χ²) was applied to evaluate differences in genotype and allele frequencies across groups.

The chi-square (χ²) test was employed to examine differences in categorical variables, while the Kruskal–Wallis H test was utilized to evaluate variations among continuous variables. A univariate logistic regression analysis was performed to identify potential predictor variables. Variables that demonstrated statistical significance (P<0.05) in the univariate analysis were subsequently entered into an binary multivariate logistic regression model to estimate the odds ratios (OR) and their corresponding 95% confidence intervals (CI) for evaluating the association between DN and the selected variables. Statistical significance was defined as a two-tailed P-value of less than 0.05. A predictive nomogram for diabetic nephropathy was developed using the regression modeling (rms) package in R software (version 4.0.2), incorporating significant independent predictors identified from multivariate analysis.

## 3 Results

### 3.1 Distribution genotypes of MTHFR C677T

This study included 397 Han Chinese participants (222 males and 175 females) from Shanxi Province in northern China, with a mean age of 60.72 ± 12.92 years. The distribution of genotypes and alleles at the C677T locus did not deviate significantly from Hardy-Weinberg equilibrium in the study population, as confirmed by chi-square testing (χ² = 2.632, P > 0.05), as shown in **Table 1**.

**Table 1:** Hardy-Weinberg equilibrium test for the MTHFR polymorphism.

MTHFR C677T	Actual frequency (%) (%)	Theoretical frequency(%)	χ^2^	P-Value
CC	91(22.92)	83(20.90)	2.632	0.1047
CT	181(45.59)	197(49.63)		
TT	125(31.49)	117(29.47)		
Total	397	397		

### 3.2 Baseline characteristics and univariate analysis results

A total of 397 patients with diabetes were enrolled in the study. The DN group included 153 individuals, while the N-DN group comprised 244. Baseline clinical characteristics of the study cohort are presented in **Table 2**. Comparative analysis between the two groups showed that patients in the DN group had a longer duration of diabetes and a greater number of individuals carrying the MTHFR 677TT genotype. Furthermore, the prevalence of comorbidities—including coronary heart disease, atherosclerosis, hypertension, peripheral vascular disease, and retinopathy was significantly higher in the DN group ((30.10% vs 18.03%, 92.16% vs 84.43%, 78.43% vs 55.74%, 66.67% vs 46.72%, 43.14% vs 12.30%, 57.52% vs 17.21%, P<0.05). Furthermore, analysis of relevant laboratory parameters demonstrated that the DN group exhibited significantly higher levels of renal function-related metabolic markers, including estimated glomerular filtration rate (eGFR), blood urea nitrogen (BUN), urinary microalbumin (Alb), urinary albumin-to-creatinine ratio (ACR), and 24-hour urinary protein (UCSF-24h), as well as homocysteine (HCY) and glycated hemoglobin (HbA1c), compared to the N-DN group (P < 0.05).

**Table 2:** Baseline characteristics and univariate analysis results of study participants.

Characteristic	N-DN Group (n =244)	DN Group (n =153)	P value
Age, years	60.30 ± 15.00	61.39.00 ± 17.00	0.418
Sex (Male) n (%)	132(54.10%)	90(58.82%)	0.406
BMI (kg/m^2^)	25.18 ± 3.98	26.84 ± 3.97	0.538
Smoking, n (%)	82(33.61%)	60(39.22%)	0.282
Drinking, n (%)	62(25.41%)	42(27.45%)	0.725
Family history of diabetes, n (%)	86(35.25%)	69(45.10%)	0.057
Duration of diabetes, years			0.000*
<5 years	82(33.60%)	25(16.34%)	
5-10 years	73(29.92%)	36(23.53%)	
11-15 years	41(16.80%)	22(14.38%)	
16-20 years	28(11.48%)	40(26.14%)	
>20 years	20(8.20%)	30(19.61%)	
Coronary heart disease, n (%)	44(18.03%)	46(30.10%)	0.007*
Cerebral infarction, n (%)	66(27.05%)	52(34.00%)	0.144
atherosclerosis, n (%)	206(84.43%)	141(92.16%)	0.029*
hypertension, n (%)	136(55.74%)	120(78.43%)	0.000*
peripheral vascular disease, n (%)	114(46.72%)	102(66.67%)	0.000*
Neuropathy, n (%)	223(91.39%)	147(96.08%)	0.100
retinopathy, n (%)	30(12.30%)	66(43.14%)	0.000*
MTHFR C677T			0.000*
CC	95(38.93%)	13(8.50%)	
CT	107(43.85%)	52(33.99%)	
TT	42(17.21%)	88(57.52%)	
HCY (μmmol/L)	13.90 ± 4.67	15.03 ± 7.85	0.000*
HCY			
≤15 (μmmol/L)	195(79.92%)	89(58.17%)	0.000*
16-30 (μmmol/L)	38(15.57%)	53(34.64)
>30 (μmmol/L)	11(4.51%)	11(7.19%)
ALT (U/L)	23.15 ± 9.75	21.35 ± 10.10	0.316
AST (U/L)	22.39 ± 8.00	22.21 ± 9.10	0.048*
eGFR [mL/(min*1.73m^2^]	104.97 ± 38.70	91.47 ± 57.69	0.000*
BUN (mmo/L)	5.43 ± 2.06	7.45 ± 3.45	0.000*
TC (mmo/L)	4.48 ± 1.58	5.08 ± 1.47	0.160
TG (mmo/L)	1.84 ± 1.15	2.62 ± 1.30	0.077
LDL (mmo/L)	2.45 ± 1.06	2.58 ± 0.95	0.177
HDL ((mmo/L)	1.90 ± 0.38	1.23 ± 0.32	0.002*
HOMA-IR	4.25 ± 2.43	5.86 ± 3.12	0.001*
HbA1c (%)	8.56 ± 2.77	9.15 ± 2.95	0.001*
UCSF 24h (g/24H)	0.67 ± 0.84	1.46 ± 1.37	0.000*
Alb (mg/L)	50.50 ± 23.16	306.86 ± 188.85	0.000*
ACR (mg/g)	107.52 ± 120.79	429.24 ± 342.10	0.000*

*Comparison of two groups, *P* < 0.05.

### 3.3 MTHFR C677T gene polymorphism and diabetic nephropathy

To further elucidate the role of MTHFR C677T gene polymorphism in diabetic nephropathy, we performed a stratified analysis according to the different MTHFR C677T genotypes. Statistical results demonstrated a significant association between the MTHFR C677T genotype and diabetic nephropathy, with a higher prevalence of the TT genotype observed in patients with diabetic nephropathy. Renal function–related metabolic parameters, including estimated glomerular filtration rate (eGFR), blood urea nitrogen (BUN), urinary microalbumin (Alb), and urinary microalbumin-to-creatinine ratio (ACR), exhibited significant differences across the three genotypic groups. The TT genotype was associated with markedly impaired renal function. These findings further support an association between the MTHFR C677T polymorphism and renal dysfunction in patients with diabetes mellitus, as presented in **Table 3**.

**Table 3:** Comparison of MTHFR C677T genotypes in the study population.

Characteristic	MTHFR C677T genotypes			P value
	CC	CT	TT	
Sex				
Male (%)	59(26.58%)	94(42.34%)	69(31.08%)	0.560
Female (%)	49(28.00%)	65(37.14%)	61(34.86%)	
N-DN Group (%)	95 (38.94%)^a,b,c^	107 (43.85)% ^a,b,c^	42 (17.21)% ^a,b,c^	0.000*
DN Group (%)	13 (8.50%) ^a,b,c^	52 (33.99%) ^a,b,c^	88 (57.51%)^a,b,c^	0.000*
Coronary heart disease, n (%)	13(14.44%)^a^	37(41.12%)	40(44.44%)^a^	0.003*
Cerebral infarction, n (%)	27 (22.89%)	48(40.68%)	43(36.44%)	0.393
hypertension, n (%)	47(18.36%)^a^	90(35.16%)^c^	119(46.48%)^a,c^	0.000*
AST (U/L)	21.37 ± 7.05	22.60 ± 10.00	22.78 ± 7.88	0.781
ALT (U/L)	22.02 ± 10.00	22.70 ± 9.30	22.51 ± 10.70	0.526
eGFR [mL/(min*1.73m^2^]	107.01 ± 39.81 ^a^	101.06 ± 45.22 ^c^	92.19 ± 51.89 ^a,c^	0.000*
BUN (mmo/L)	5.87 ± 1.73^a^	5.82 ± 2.60 ^c^	6.97 ± 2.65^a,c^	0.002*
HOMAIR	4.59 ± 2.88	4.61 ± 2.48	5.43 ± 2.97	0.208
TC (mmo/L)	4.69 ± 1.43	4.67 ± 1.48	4.77 ± 1.46	0.172
TG (mmo/L)	1.94 ± 1.28	2.33 ± 1.04	2.06 ± 1.27	0.904
LDL-C (mmo/L)	2.58 ± 1.04	2.51 ± 0.95	2.41 ± 0.98	0.200
HbA1c (%)	8.80 ± 3.17	8.82 ± 3.10	8.73 ± 2.43	0.981
UCSF 24h (g/24H)	0.73 ± 0.91	0.95 ± 1.02	1.21 ± 1.06	0.224
Alb (mg/L)	56.70 ± 38.55 ^a^	130.13 ± 109.30 ^c^	249.68 ± 219.40 ^a,c^	0.000*
ACR (mg/g)	125.47 ± 149.34 ^a^	206.51 ± 233.50	350.17 ± 293.24 ^a^	0.011*

*Comparison of three groups, *P* < 0.05. ^a^Compared with TT genotype, P<0.05. ^b^Compared with TT genotype, P<0.05. ^c^Compared with TT genotype, P<0.05.

**Figure 2:**
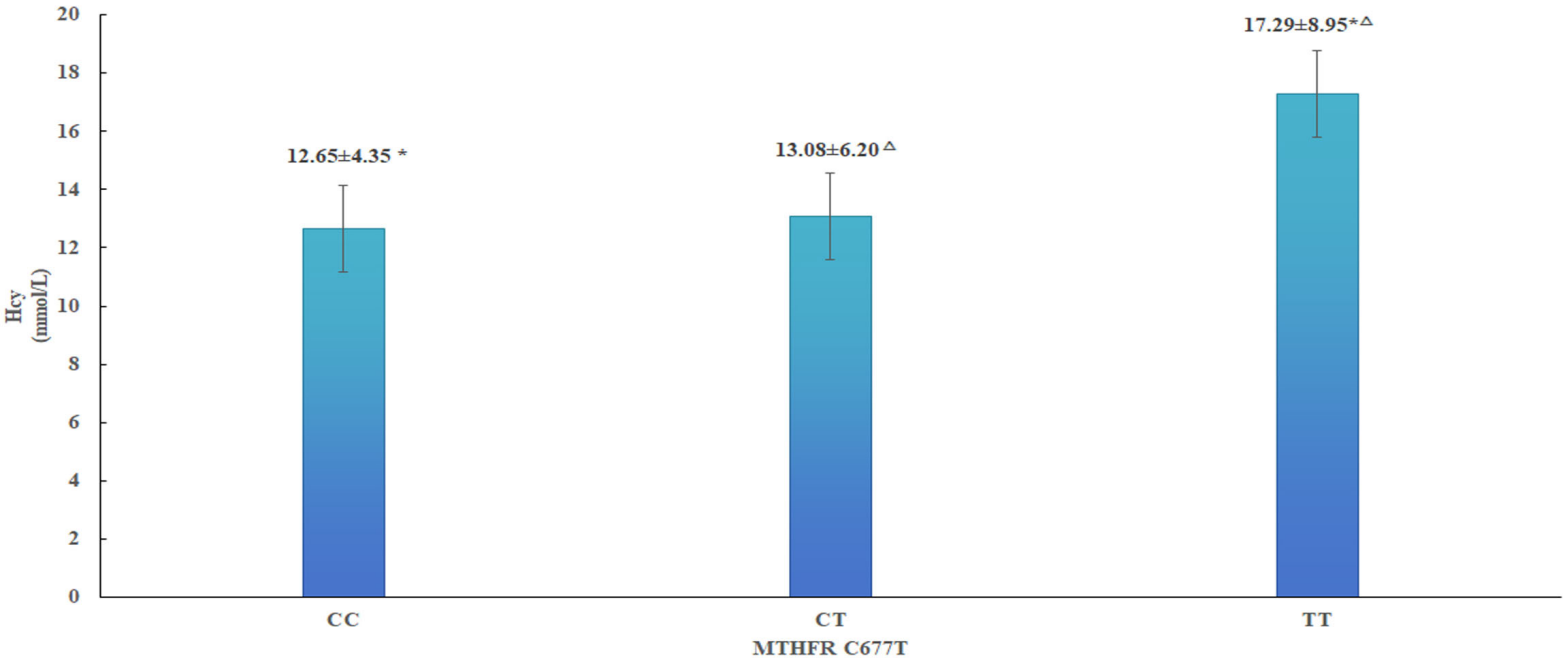
Plasma Hcy level with different genotypes of MTHFR C677T. HCY levels were significantly different among the three groups (P<0.001). */△: Compared with TT genotype, P<0.001.

The MTHFRC677T gene polymorphism is closely associated with homocysteine (Hcy) metabolism in the human body. Elevated homocysteine (HHcy) levels have been well established as a risk factor for cardiovascular and cerebrovascular diseases. To investigate whether the association between the MTHFR C677T genotype and diabetic nephropathy is mediated by Hcy levels. We analyzed plasma homocysteine concentrations across different genotypic groups and found that individuals carrying the 677TT genotype had significantly higher circulating homocysteine concentrations (17.29 ± 8.95 μmol/L) compared to those with the 677CT (13.08 ± 6.20 μmol/L) and 677CC (12.65 ± 4.35 μmol/L) genotypes (P < 0.05), as shown in **Figure 2**. This further elucidates the role of the MTHFR C677T gene–homocysteine–diabetic nephropathy pathway in the pathogenesis of diabetic nephropathy.

### 3.4 Prediction of risk factors for diabetic nephropathy

To identify risk factors for diabetic nephropathy, in addition to the MTHFR C677T genotype and homocysteine (Hcy), this study further selected two sets of variables exhibiting significant differences. Binary multivariate logistic regression analysis was subsequently performed to determine the independent risk factors associated with diabetic nephropathy in patients with diabetes. The results indicated that, after adjustment for confounding factors, the risk of diabetic nephropathy (DN) among carriers of the 677TT and 677CT genotypes was 3.298-fold and 12.713-fold higher, respectively, compared to carriers of the 677CC genotype. In addition, each one standard deviation increase in diabetic peripheral vascular disease, diabetic retinopathy, triglycerides (TG), HOMA-IR, blood urea nitrogen (BUN), and urinary albumin-to-creatinine ratio (ACR) was associated with an increased risk of diabetic nephropathy (OR = 2.462, 4.572, 1.548, 1.133, 1.254, 1.003, respectively). See **Table 4**.

**Table 4:** Logistic regression analysis of risk factors of diabetic nephropathy.

Significant variables	OR	95% CI	P value
Peripheral vascular disease	2.462	1.304-4.755	0.006
Retinopathy	4.572	2.319-9.257	<0.001
TG	1.548	1.228-2.038	<0.001
HOMA-IR	1.133	1.037-1.269	0.013
BUN	1.254	1.082-1.480	0.006
ACR	1.003	1.001-1.005	<0.001
MTHFR C677T genotype			
CC	–		
CT	3.298	1.443-7.985	0.006
TT	12.713	5.223-33.371	0.005
HCY			
≤15μmol/L	–		
16-30μmol/L	1.571	0.406-4.592	0.202
>30μmol/L	1.379	1.228-2.-38	0.509

**Figure 3:**
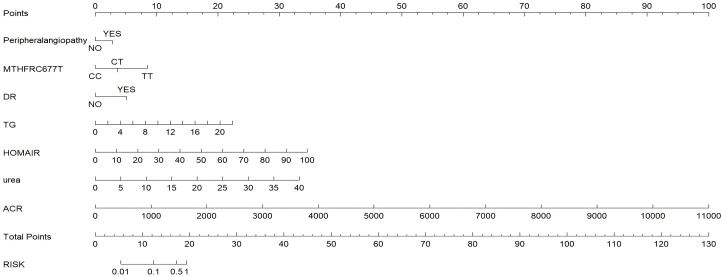
Nomogram calculator of the significant factors in prediction for diabetic nephropathy.

**Figure 4:**
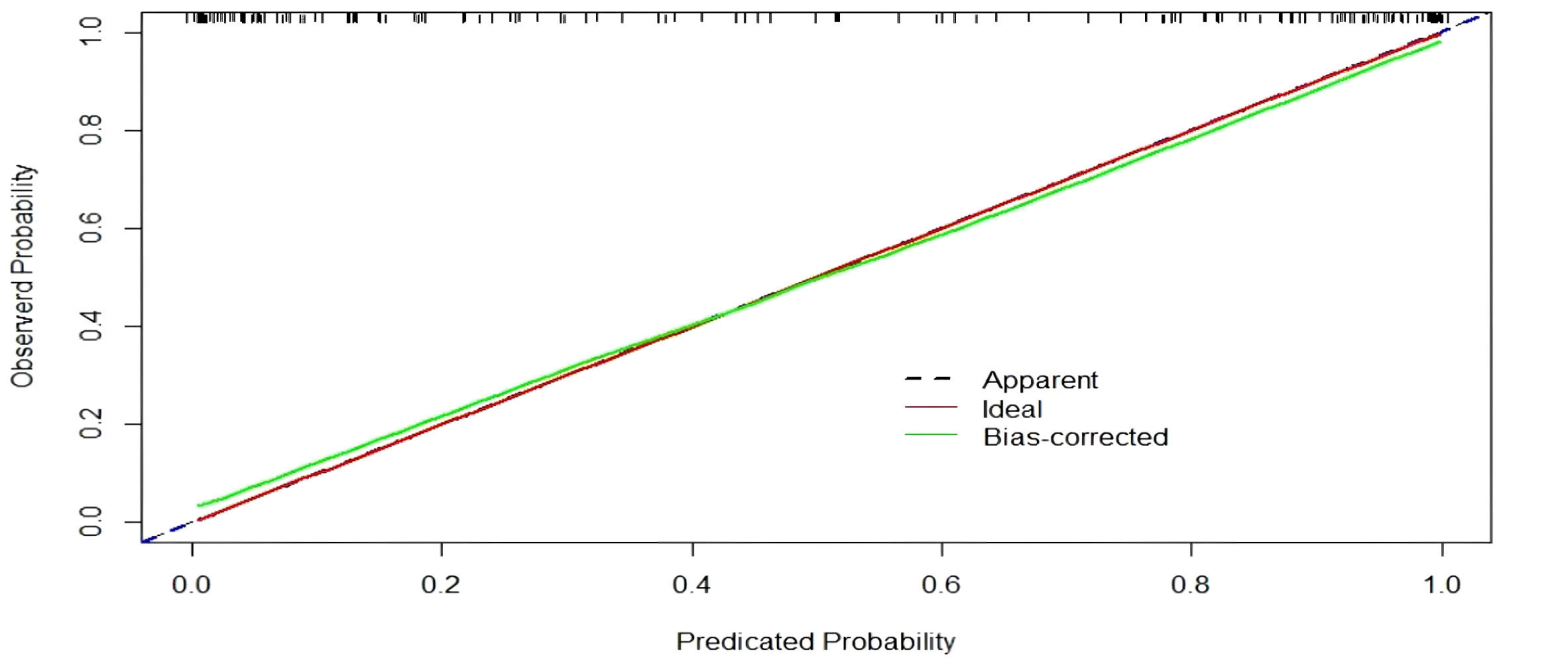
Calibration curve for model validation.

**Figure 5:**
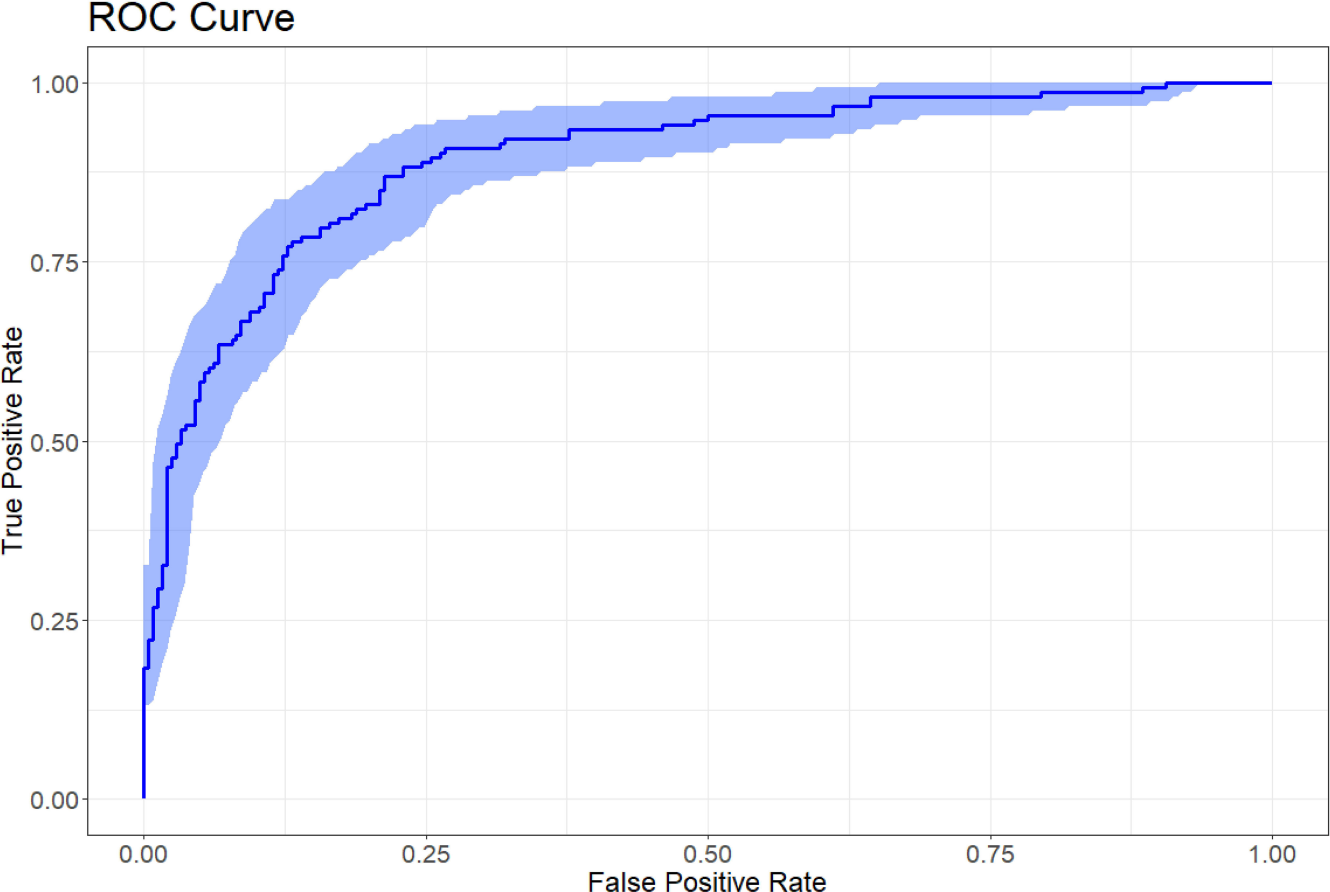
ROC curve of nomogram model.

**Figure 6:**
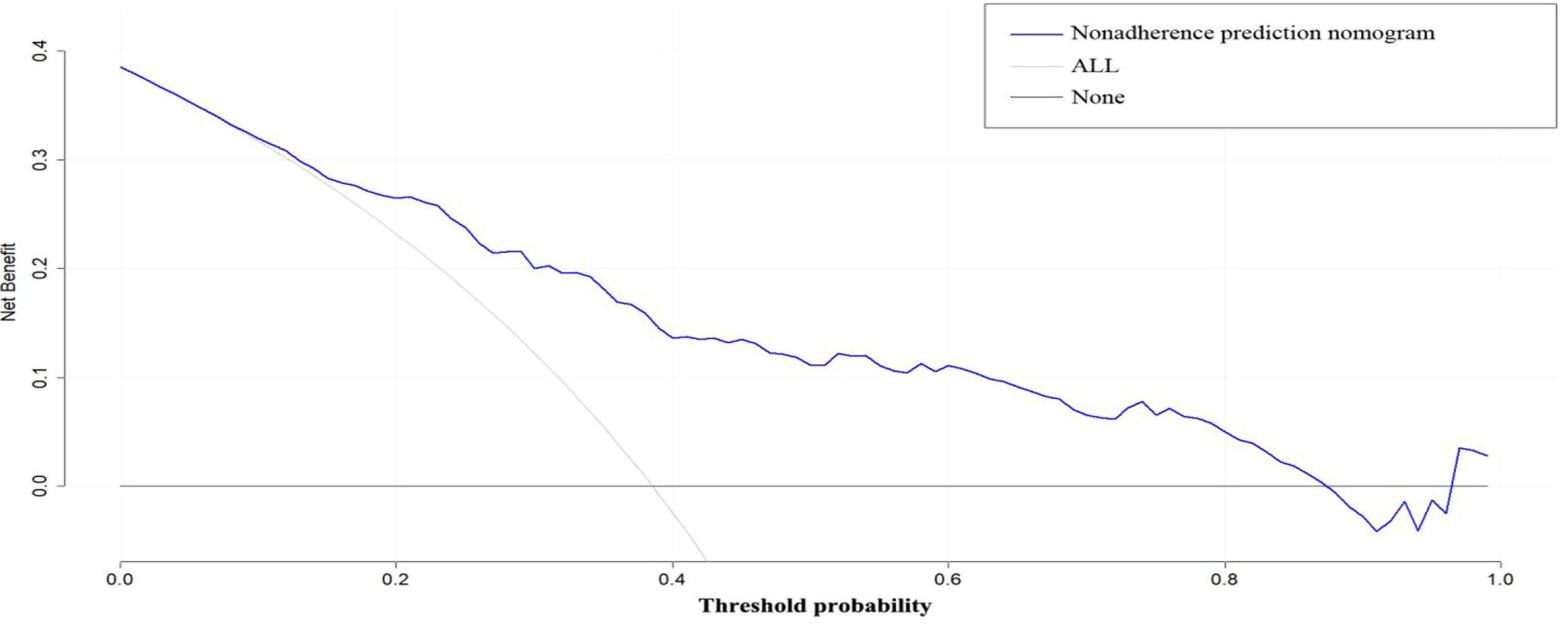
Decision curve of nomogram model.

A nomogram was developed to visualize the predictive model for factors significantly associated with the risk of diabetic nephropathy onset, as shown in **Figure 3**. The nomogram model was internally validated using 1,000 Bootstrap resamples. The C-index for predictive accuracy was 0.906 (P < 0.01), indicating high discriminative ability. A calibration curve was constructed, which closely aligned with the ideal diagonal line, demonstrating excellent agreement between predicted and observed probabilities and confirming good model performance. See **Figure 4**. The receiver operating characteristic (ROC) curve for the internal validation of the nomogram model is presented in **Figure 5**. The results demonstrate that the nomogram achieves an area under the curve (AUC) of 0.906 (95% confidence interval [CI]: 0.8611–0.9269, P < 0.01) in predicting diabetic nephropathy. The decision curve analysis is displayed in **Figure 6**, indicating that the model yields a higher net benefit across threshold probabilities ranging from 0.12 to 0.87, compared to both treat-all and treat-none strategies.

## 4 Discussion

Diabetic nephropathy, a prominent microvascular complication associated with diabetes mellitus, is a key contributor to elevated rates of morbidity and mortality, as well as shortened life expectancy in patients with type 2 diabetes. Following its onset, DN exacerbates the advancement of disease, induces significant impairment in kidney function, and can progress to end-stage renal disease or uremia, thereby placing considerable physical and emotional strain on affected individuals and substantially diminishing their quality of life. In the present study, DN was identified in 153 of the 397 participants diagnosed with type 2 diabetes, corresponding to a prevalence of 38.54%. This rate is notably higher than documented by Zhang XX (14) in Chinese cohorts but aligns closely with data reported by Afkarian M (15) and De Boer IH (16) in populations from the United States. These findings emphasize the heightened susceptibility to diabetic nephropathy among diabetic individuals and reinforce the necessity for early risk assessment and prompt preventive strategies.

Our previous findings demonstrated that, despite a dietary pattern in the Shanxi region conducive to lowering homocysteine levels, the high prevalence of hyperhomocysteinemia remains strongly associated with the MTHFR C677T gene polymorphism, which contributes to the elevated incidence and increased severity of H-type hypertension in this population (13). Emerging evidence indicates a significant association between elevated plasma homocysteine (Hcy) levels and the development or progression of type 2 diabetic nephropathy. This study further investigated the association among the MTHFR C677T gene polymorphism, homocysteine levels, and diabetic nephropathy in the target population. The findings indicate that the MTHFR C677T polymorphism is significantly associated with elevated circulating homocysteine concentrations and an increased risk of diabetic nephropathy in patients with diabetes mellitus. The MTHFR C677T variant is more prevalent in individuals with diabetic nephropathy and is associated with elevated plasma homocysteine levels as well as poorer renal function-related metabolic parameters. The MTHFR C677T gene mutation, which leads to increased circulating homocysteine concentrations, is significantly linked to a higher risk of developing diabetic nephropathy in patients with diabetes mellitus. These findings suggest that the MTHFR C677T gene polymorphism and homocysteine (Hcy) may serve as potential predictive markers for diabetic nephropathy (DN). Regular monitoring of serum Hcy levels in patients with diabetes mellitus may facilitate the early detection of DN and help prevent the onset and progression of renal injury. The underlying mechanisms of homocysteine (Hcy)-related diabetic nephropathy (DN) injury remain incompletely understood. It is hypothesized that elevated Hcy levels may contribute to renal damage by inducing endothelial dysfunction, promoting cellular proliferation, increasing oxidative stress, and establishing a pro-thrombotic state—leading to microvascular endothelial injury. Additionally, Hcy may directly affect glomerular mesangial cells, triggering sclerosis and reducing plasma and tissue adenosine levels (17, 18). Furthermore, emerging evidence suggests that hyperhomocysteinemia may induce renal injury through the activation of ferroptosis pathways (19). Hcy can also induce ferroptosis in endothelial cells via the Xc-/GPX4 signaling pathway (20). It is hypothesized that elevated levels of Hcy may contribute to renal injury by triggering the ferroptosis pathway (21).

Furthermore, given the complex pathogenesis of diabetic nephropathy (DN), multiple risk factors beyond the MTHFR C677T gene polymorphism and homocysteine (Hcy) levels may be involved. Therefore, we conducted a comprehensive evaluation and analysis of potential risk factors associated with DN and attempted to establish a predictive model for assessing the risk of diabetic nephropathy. Therefore, we performed a forward multivariate logistic regression analysis. The results indicated that, in addition to the MTHFR C677T gene polymorphism and homocysteine (Hcy) levels, diabetic peripheral vascular disease, diabetic retinopathy, triglycerides (TG), HOMA-IR, blood urea nitrogen (BUN), and urinary albumin-to-creatinine ratio (ACR) were significantly associated with an increased risk of developing diabetic nephropathy. Incorporating these factors into risk assessment may enhance the prediction of future DN onset in patients with diabetes. The model demonstrated favorable calibration and strong discriminative ability.

Peripheral vascular disease is a prevalent macrovascular complication in individuals with diabetes, characterized primarily by endothelial dysfunction, dyslipidemia, and atherosclerosis. Diabetic nephropathy, a microvascular complication of diabetes, is predominantly defined by basement membrane thickening and microcirculatory disturbances associated with thrombosis. Accumulating evidence has demonstrated a significant association between diabetic macrovascular and microvascular complications. Diabetic patients with peripheral vascular disease exhibit a substantially increased risk of glomerular filtration rate (GFR) decline (22, 23). This is consistent with our research findings indicating that peripheral vascular lesions constitute a significant risk factor for the development of diabetic nephropathy. Diabetic retinopathy and diabetic nephropathy are both microvascular complications of diabetes mellitus. They exhibit structural and functional similarities in their underlying tissues; furthermore, research indicates that their shared pathogenic mechanisms often lead to concurrent clinical presentation (24–27). Patients with type 2 diabetes who have retinopathy are at increased risk of renal function impairment and exhibit a higher likelihood of developing diabetic nephropathy (28–30). This suggests that diabetic retinopathy may serve as a significant predictor for the onset of diabetic nephropathy.

Triglycerides (TG) are among the primary forms of lipids in the human body. In patients with diabetes, chronic disturbances in glucose metabolism and dyslipidemia represent key contributors to the development of diabetic nephropathy (DN) (31, 32). Elevated lipid levels can induce vascular endothelial injury, promote atherosclerotic plaque formation, increase blood viscosity, impair microcirculation, and ultimately lead to vascular fibrosis and thrombosis, thereby contributing to renal dysfunction (33, 34). In addition, elevated triglyceride (TG) levels may induce chronic inflammation and cellular oxidative stress, thereby promoting the secretion of adipokines from adipocytes and impairing glomerular and renal tubular function. Moreover, dyslipidemia contributes to the development of insulin resistance (IR) in patients with diabetes. Research indicates that IR and renal function decline are mutually reinforcing (35, 36). IR can lead to kidney injury through impaired renal hemodynamics, glomerular mesangial expansion, and renal tubulointerstitial fibrosis. Conversely, as renal function deteriorates, metabolic acidosis, systemic inflammation, oxidative stress, and endothelial dysfunction further exacerbate insulin resistance (37). This study further demonstrates that triglycerides (TG) and HOMA-IR are independent risk factors for the development of diabetic nephropathy.

Serum urea nitrogen (BUN) and the urinary albumin-to-creatinine ratio (ACR) are widely recognized clinical biomarkers for the assessment of renal function (38, 39). Blood urea nitrogen (BUN) is a primary end product of protein metabolism and is predominantly eliminated via renal excretion. Impairment of renal function leads to a corresponding elevation in BUN levels. The urinary albumin-to-creatinine ratio (ACR) exhibits high stability and provides a more accurate assessment of early renal injury in patients with diabetes compared to traditional random measurements of urinary microalbumin (40–42). The predictive model developed in this study incorporates both markers to improve the accuracy of risk prediction for diabetic nephropathy.

This study has several limitations. First, the study population was recruited from a single tertiary hospital, and regional variations in healthcare-seeking behavior were not accounted for, which may introduce selection bias. Second, due to the unavailability of suitable external datasets, the predictive model was validated only through internal resampling methods. Future studies should aim to validate and replicate the model using high-quality external data, with the goal of refining the risk assessment algorithm to achieve greater accuracy and enhance its clinical utility.

## 5 Conclusions

Our findings demonstrate that the MTHFR C677T gene polymorphism is associated with elevated circulating homocysteine levels and an increased risk of diabetic nephropathy among Chinese patients with diabetes. Furthermore, we developed a robust risk prediction model incorporating the MTHFR C677T genotype, homocysteine, diabetic peripheral vascular disease, diabetic retinopathy, triglycerides (TG), HOMA-IR, blood urea nitrogen (BUN), and urinary albumin-to-creatinine ratio (ACR), which enables early prediction and prevention of diabetic nephropathy.

## Data Availability

The original contributions presented in the study are included in the article/supplementary material/Further inquiries can be directed to the corresponding author.
